# “I Haven’t Been Diagnosed, but I Should Be”—Insight Into Self-diagnoses of Common Mental Health Disorders: Cross-sectional Study

**DOI:** 10.2196/39206

**Published:** 2023-01-13

**Authors:** Lauren A Rutter, Jacqueline Howard, Prabhvir Lakhan, Danny Valdez, Johan Bollen, Lorenzo Lorenzo-Luaces

**Affiliations:** 1 Department of Psychological and Brain Sciences Indiana University Bloomington Bloomington, IN United States; 2 Department of Applied Health Science School of Public Health Indiana University Bloomington Bloomington, IN United States; 3 Luddy School of Informatics, Computing, and Engineering Indiana University Bloomington Bloomington, IN United States

**Keywords:** assessment, depression, anxiety, self-report, social media

## Abstract

**Background:**

In recent years, social media has become a rich source of mental health data. However, there is a lack of web-based research on the accuracy and validity of self-reported diagnostic information available on the web.

**Objective:**

An analysis of the degree of correspondence between self-reported diagnoses and clinical indicators will afford researchers and clinicians higher levels of trust in social media analyses. We hypothesized that self-reported diagnoses would correspond to validated disorder-specific severity questionnaires across 2 large web-based samples.

**Methods:**

The participants of study 1 were 1123 adults from a national Qualtrics panel (mean age 34.65, SD 12.56 years; n=635, 56.65% female participants,). The participants of study 2 were 2237 college students from a large university in the Midwest (mean age 19.08, SD 2.75 years; n=1761, 75.35% female participants). All participants completed a web-based survey on their mental health, social media use, and demographic information. Additionally, the participants reported whether they had ever been diagnosed with a series of disorders, with the option of selecting “Yes”; “No, but I should be”; “I don’t know”; or “No” for each condition. We conducted a series of ANOVA tests to determine whether there were differences among the 4 diagnostic groups and used post hoc Tukey tests to examine the nature of the differences.

**Results:**

In study 1, for self-reported mania (*F*_3,1097_=2.75; *P*=.04), somatic symptom disorder (*F*_3,1060_=26.75; *P*<.001), and alcohol use disorder *(F*_3,1097_=77.73; *P*<.001), the pattern of mean differences did not suggest that the individuals were accurate in their self-diagnoses. In study 2, for all disorders but bipolar disorder (*F*_3_,_659_=1.43; *P*=.23), ANOVA results were consistent with our expectations. Across both studies and for most conditions assessed, the individuals who said that they had been diagnosed with a disorder had the highest severity scores on self-report questionnaires, but this was closely followed by individuals who had not been diagnosed but believed that they should be diagnosed. This was especially true for depression, generalized anxiety, and insomnia. For mania and bipolar disorder, the questionnaire scores did not differentiate individuals who had been diagnosed from those who had not.

**Conclusions:**

In general, if an individual believes that they should be diagnosed with an internalizing disorder, they are experiencing a degree of psychopathology similar to those who have already been diagnosed. Self-reported diagnoses correspond well with symptom severity on a continuum and can be trusted as clinical indicators, especially in common internalizing disorders such as depression and generalized anxiety disorder. Researchers can put more faith into patient self-reports, including those in web-based experiments such as social media posts, when individuals report diagnoses of depression and anxiety disorders. However, replication and further study are recommended.

## Introduction

### Background

Mental disorders are one of the leading public health problems worldwide [1-3], yet they are often underdetected or misdiagnosed, particularly in underrepresented groups (sexual, gender, racial, and ethnic minorities) and young adults [4-8]. The stigma of mental illness [9], concerns about confidentiality, shame, or a variety of other reasons lead some individuals to avoid explicit help-seeking behaviors that could alleviate the symptoms of mental disorders [10]. Some turn to social media and web-based resources to share and better understand their mental health symptoms and experiences [11].

### Prior Work

In recent years, social media has become a rich source of data for studies of mental health communication, disclosure, prevalence rates, and trajectories [12]. In the medical field, more generally, data science approaches have allowed researchers to mine large health care data sets from social media [13]. In a systematic review of how social media data have been used to classify and predict the mental health state of users [14], text analysis was found to be the most common analytic method across 48 studies, in particular, the use of natural language processing and unsupervised machine learning algorithms to analyze patterns of term use. For example, ten Thij et al [15] searched on Twitter (Twitter Inc) for individuals who had reported receiving a diagnosis of depression on the platform and compared the timing of their posts with those of a random sample of Twitter users, finding differences in the pattern of activity by time of day. Specifically, the individuals who reported depression diagnoses showed more web-based activity during later hours of the day. Similarly, Bathina et al [16] showed the frequency of occurrence of cognitive distortion schema, sequences of 1 to 5 words associated with the expression of cognitive distortions, in the language of individuals who reported a clinical diagnosis of depression on Twitter versus in the language of a random sample of individuals.

Analyses of social media data, such as the works of ten Thij et al [15], Bathina et al [16], and Coppersmith et al [17], have historically relied on individuals’ self-disclosure of mental health status or search terms related to mental health disorders, for example, someone tweeting “I was just diagnosed with depression by my doctor.” A key limitation of these approaches is self-selection bias, which results, in part, from relying on people’s willingness to reveal stigmatized identities. In addition, people who are willing to self-disclose their mental health status on a public platform may be less inhibited or more likely to seek social support but may not be representative of all those who struggle with their mental health. For example, in 2014, De Choudhury and De [18] studied the self-disclosure of mental health problems on Reddit. On Reddit, individuals discuss a variety of mental health concerns, ranging from “daily grind” to highly specific questions about their diagnoses and treatment. An analysis of subreddits on “mental health,” that is, Reddit channels on specific topics, showed that individuals who are a part of subreddits related to mental health used more negative emotion words and more frequently disclosed challenges of daily activities than participants (“redditors”) in more general Reddit channels. In addition, anonymous redditors tended to be more disinhibited and share more details about their personal relationships and health issues in casual terms that did not reflect their current mental health status. In summary, anonymous Reddit accounts (“throwaway Reddit accounts”) showed greater levels of self-disclosure related to mental health and decreased feelings of vulnerability [18].

### Goal of This Work

Although text analysis is the most commonly used method to study mental disorders at scale, the reliability and validity of web-based data have been called into question, particularly with respect to self-reported diagnoses. Although we have reason to believe that individuals tend to be relatively accurate reporters of their mental health experiences [19], relying on text analysis alone may be perceived as insufficient to establish ground truth. Although clinician reports may provide an alternative to self-reports, they are more time intensive to collect at scale, require access to care, and are costly [20,21]. These concerns warrant a multimodal approach that combines language modeling, sentiment, clinical diagnostics, and user engagement to reliably study common psychiatric disorders from social media data [22,23]. Crucial to many of these efforts is the ability to triangulate self-reported diagnostic information with clinical indicators. This is the idea that motivated our study, which asked the following question: Do self-reported diagnoses correspond to validated disorder-specific severity questionnaire scores?

A better understanding of the degree of correspondence between self-reported diagnoses and clinical indicators will afford researchers and clinicians higher levels of trust in self-reported diagnoses on social media. This is particularly the case for a common approach that relies on web-based statements of self-reported diagnoses to establish clinically relevant samples [15-18].

The primary aim of this study was to examine whether people are “accurate” when they report that they are or should be diagnosed with a mental disorder. In other words, does an individual’s belief that they should be diagnosed with a disorder correspond to their scores on validated self-report measurements of their symptoms. We addressed this research question using two large-scale samples of thousands of individuals: (1) a nationally representative sample of adults and (2) a large sample of university undergraduates. The participants were asked to fill out self-report questionnaires on a variety of mental disorder symptoms and asked whether they had ever been diagnosed with any conditions or felt that they should be diagnosed with those. We first hypothesized that there would be a positive relationship between self-reported disorder-specific severity scores and having received a diagnosis. Second, we expected that there would be significant differences between the participants who believed that they should receive a diagnosis and those who reported that they had never been diagnosed. Finally, we hypothesized that the group of participants who believed that they should be diagnosed would look more similar to the participants who had been diagnosed than to those who had never been diagnosed, lending initial credibility and support for relying on self-diagnoses in future research.

## Methods

### Ethics Approval

This study was approved by the institutional review board of Indiana University (#2002549202 and #2005948214).

### Recruitment

The participants were recruited for a broader study called the Survey Online Cohorts for Internalizing Symptoms and Language. The aim of the Survey Online Cohorts for Internalizing Symptoms and Language studies was to triangulate social media data with self-reported mental health status. In this manuscript, we focused on the latter source of data. The participants were sampled from two distinct sources: (1) a national Qualtrics panel of Twitter users (study 1) and (2) a psychology student pool at a large Midwestern university in the United States, the participants from which received credit for participation (study 2). In both studies, the participants completed a battery of questionnaires entirely on the web. Our inclusion criteria consisted of passing reCAPTCHA and affirming “Yes” to the question, “I will provide my best answers.” After excluding the individuals who could not pass the reCAPTCHA or did not commit to providing truthful answers, a total of 3460 participants responded to our surveys. Although the procedural elements and questionnaires completed by the participants were nearly identical, we have first presented the nationally representative sample of Qualtrics participants (study 1) and then presented the college student sample (study 2). Data were collected between July 2020 and April 2022. Additional data collection is ongoing for facets of a larger project unrelated to this study.

### Participants

The demographic information of the participants of studies 1 and 2 is presented in Table 1. For study 1 (n=1123), our sample was well represented, with the participants’ racial demographics mapped onto census data and a large distribution of age. As for study 2 (n=2337), it can be assumed that the sample was not nationally representative, as most participants were Indiana residents who were college aged.

**Table 1:** Demographic information of a web-based panel (study 1; n=1123) and university students (study 2; n=2337) who responded to a web-based survey on mental health and social media use (Survey Online Cohorts for Internalizing Symptoms and Language study).

Variable		Value	
		Study 1	Study 2
Age (years), mean (SD)		34.65 (12.56)	19.08 (2.75)
**Race, n (%)**			
	White	826 (73.68)	1645 (70.3)
	Black	128 (11.42)	170 (7.26)
	American Indian or Alaska Native	7 (0.62)	2 (0.08)
	Asian	62 (5.53)	240 (10.27)
	Native Hawaiian or Pacific Islander	1 (0.09)	2 (0.08)
	Hispanic	86 (7.67)	123 (5.26)
	Other	11 (0.98)	39 (1.67)
**Sex, n (%)**			
	Male	484 (43.18)	491 (21.01)
	Female	635 (56.65)	1761 (75.35)
	Prefer not to answer	2 (0.18)	2 (0.09)
**Gender, n (%)**			
	Man	492 (43.89)	491 (21.01)
	Woman	615 (54.86)	1732 (74.11)
	Nonbinary	12 (1.07)	25 (1.07)
	Other	2 (0.18)	2 (0.08)
	Prefer not to answer	0 (0)	4 (0.17)
**Sexual orientation, n (%)**			
	Heterosexual	921 (82.16)	1896 (81.13)
	Homosexual	42 (3.75)	52 (2.23)
	Bisexual or pansexual or both	124 (11.06)	226 (9.67)
	Other	18 (1.61)	62 (2.65)
	Prefer not to answer	16 (1.43)	18 (0.77)
**Annual household income (US $), n (%)**			
	<10,000	99 (8.83)	116 (4.96)
	10,000-19,999	95 (8.47)	68 (2.91)
	20,000-29,999	141 (12.58)	94 (4.02)
	30,000-39,999	103 (9.19)	105 (4.49)
	40,000-49,999	65 (5.8)	102 (4.36)
	50,000-59,999	79 (7.05)	106 (4.53)
	60,000-69,999	51 (4.55)	113 (4.83)
	70,000-79,999	73 (6.51)	107 (4.58)
	80,000-89,999	38 (3.39)	130 (5.56)
	90,000-99,999	64 (5.71)	164 (7.01)
	100,000-149,999	171 (15.25)	469 (20.04)
	>150,000	142 (12.67)	656 (28.07)

### Measures

#### Overview

As noted above, the measures of relevance in this research were the same for studies 1 and 2. All the study measures were self-report instruments administered on the web. Most measures, detailed below, were selected from the American Psychiatric Association (APA)’s “emerging measures” for research and clinical evaluation and are in the public domain to be used by researchers and clinicians. These web-based assessment measures are included in Section III of the Diagnostic and Statistical Manual for Mental Disorders (*DSM)*–5^th^ Edition [24]*.*

#### Insomnia Severity Index

The Insomnia Severity Index [25] is a 7-item self-report measure that assesses insomnia symptom severity over the previous 2 weeks. The items are rated on a 5-point Likert scale ranging from 0 (“none”) to 4 (“very severe”), yielding total scores of 0 to 28. The total score is interpreted as follows: 0 to 7 indicate the absence of insomnia, 8 to 14 indicate subthreshold insomnia, 15 to 21 indicate moderate insomnia, and 22 to 28 indicate severe insomnia. Prior studies have found adequate psychometric properties for the English and French versions [25-27] and indicate that the measure is likely clinically useful as a screening device [25]. In our samples, Cronbach alphas were .87 for study 1 and .86 for study 2, suggesting that this measure was internally consistent in both studies.

#### Severity Measure for Somatic Symptoms—Adult (APA, 2013)

The severity measure for somatic symptoms was adapted from the Patient Health Questionnaire Physical Symptoms—15 [28] and consists of 15 items rated on a 3-point Likert scale ranging from 0 (“not bothered at all”) to 2 (“bothered a lot”). The participants are asked to rate their somatic symptoms over the past 7 days. The total scores range from 0 to 30, with higher scores indicating more severe somatic symptoms. The scores are interpretated in terms of how they relate to the level of somatic symptom severity: 0 to 4 indicate a minimal level of severity, 5 to 9 indicate a low level of severity, 10 to 14 indicate a medium level of severity, and 15 to 30 indicate a high level of severity. In our samples, Cronbach alphas were .89 for study 1 and .81 for study 2, suggesting that this measure was internally consistent in both studies.

#### Severity Measure for Depression—Adult (APA, 2013)

The severity measure for depression was adapted from the Patient Health Questionnaire-9 (PHQ-9) [29] and consists of 9 items rated on a 4-point Likert scale ranging from 0 (“not at all”) to 3 (“nearly every day”). The participants were asked to rate their symptoms of depression over the last 7 days. Of note, we also used the PHQ-9 in examining the depressive symptoms of bipolar disorder. The PHQ-9 has excellent psychometric properties [29]. A score of 5 is considered mild, and a score of 10 has shown to maximize the sensitivity and specificity for screening for the diagnosis of depression [30]. In our samples, Cronbach alphas were .91 for study 1 and .88 for study 2, suggesting that this measure was internally consistent in both studies.

#### Severity Measure for Social Anxiety Disorder (Social Phobia)—Adult (APA, 2013)

The severity measure for social phobia symptoms consists of 10 items rated on a 5-point Likert scale ranging from 0 (“never”) to 4 (“all of the time”). The participants are asked to rate the frequency of fear and avoidance of social situations over the past 7 days. The total scores range from 0 to 40, with higher scores indicating a greater severity of social anxiety disorder (social phobia). The average score (total raw score/number of items answered) can be used as a proxy for social phobia severity: 0 indicates none, 1 indicates mild, 2 indicates moderate, 3 indicates severe, and 4 indicates extreme. In our samples, Cronbach alphas were .95 for study 1 and .93 for study 2, suggesting that this measure was internally consistent in both studies.

#### Severity Measure for Panic Disorder—Adult (APA, 2013)

The severity measure for panic disorder consists of 10 items rated on a 5-point Likert scale ranging from 0 (“never”) to 4 (“all of the time”). The participants are asked to rate the frequency of fear and avoidance of panic attacks over the past 7 days. The total scores range from 0 to 40, with higher scores indicating a greater severity of panic disorder symptoms. The average score (total raw score/number of items answered) can be used as a proxy for panic disorder severity: 0 indicates none, 1 indicates mild, 2 indicates moderate, 3 indicates severe, and 4 indicates extreme. In our samples, Cronbach alphas were .96 for study 1 and .94 for study 2, suggesting that this measure was internally consistent in both studies.

#### Severity Measure for Generalized Anxiety Disorder—Adult (APA, 2013)

The severity measure for generalized anxiety disorder (GAD) consists of 10 items rated on a 5-point Likert scale ranging from 0 (“never”) to 4 (“all of the time”). The participants are asked to rate the frequency of worry and associated symptoms over the past 7 days. Total scores range from 0 to 40, with higher scores indicating a greater severity of GAD symptoms. The average score (total raw score/number of items answered) can be used as a proxy for GAD severity: 0 indicates none, 1 indicates mild, 2 indicates moderate, 3 indicates severe, and 4 indicates extreme. In our samples, Cronbach alphas were .95 for study 1 and .91 for study 2, suggesting that this measure was internally consistent in both studies.

#### Severity Measure for Agoraphobia—Adult (APA, 2013)

The severity measure for agoraphobia consists of 10 items rated on a 5-point Likert scale ranging from 0 (“never”) to 4 (“all of the time”). The participants are asked to rate the frequency of fear and avoidance in agoraphobic situations over the past 7 days. Total scores range from 0 to 40, with higher scores indicating a greater severity of agoraphobia symptoms. The average score (total raw score/number of items answered) can be used as a proxy for agoraphobia severity: 0 indicates none, 1 indicates mild, 2 indicates moderate, 3 indicates severe, and 4 indicates extreme. In our samples, Cronbach alphas were .96 for study 1 and .93 for study 2, suggesting that this measure was internally consistent in both studies.

#### Alcohol Use Disorders Identification Test

The Alcohol Use Disorders Identification Test (AUDIT) is a 10-item self-report measure developed by the World Health Organization (1989) to assess alcohol use and misuse. The participants answer the first 8 items on a 5-point Likert scale ranging from 0 (“never”) to 4 (“daily or almost daily”) and the last 2 items on a 3-point scale (“No”’ “Yes, but not in the last year”; “Yes, during the last year”). The AUDIT assesses 2 domains of alcohol use: consumption (first 3 items) and alcohol-related problems (last 7 items). Previous studies have shown that this measure has good reliability and validity among college students [31]. A score of ≥8 indicates hazardous or harmful use of alcohol. In our sample, Cronbach alphas were .93 for study 1 and .80 for study 2, suggesting that this measure was internally consistent in both studies.

#### Substance Use—Adult (APA, 2013)

This measure was adapted from the National Institute on Drug Abuse-Modified Assist to assess the misuse of prescription medications and illicit substance use in adults aged ≥18 years. Each item asks the participant to rate the severity of their substance use during the past 2 weeks. The scale asks about 10 categories of drugs, and each item is rated on a 5-point scale ranging from 0 (“not at all”) to 4 (“nearly every day”). Rating multiple items >0 indicates a greater severity and complexity of substance use problems.

#### Altman Self-rating Mania Scale

The Altman Self-rating Mania Scale (ASRM; [32]) is a 5-item self-report scale that assesses the presence and severity of mania symptoms over the past week. Items are rated on a 5-point Likert-style scale, with response categories having different anchors depending on the symptoms. The scores range from 5 to 25, with higher scores indicating more severe manic symptoms. The interpretation of the ASRM is as follows: (1) a score of ≥6 indicates a high probability of a manic or hypomanic condition, (2) a score of ≥6 may indicate the need for treatment or further diagnostic assessment, and (3) a score of ≤5 is less likely to be associated with significant symptoms of mania. In our samples, Cronbach alphas were .84 for study 1 and .71 for study 2, suggesting that this measure was internally consistent in both studies, with good consistency in study 1 and acceptable consistency in study 2.

#### Diagnosis Questionnaire

We assessed the diagnoses of each of the disorders listed above and some additional diagnoses (insomnia, substance use disorder, depression, specific phobia, social anxiety disorder, panic disorder, posttraumatic stress disorder, GAD, agoraphobia, somatic symptom disorder, alcoholism, and mania) with the prompt, “Have you ever been clinically diagnosed with [blank] disorder?” Response options were as follows: (1) “Yes”; (2) “No, but I should be”; (3) “I don’t know”; and (4) “No.”

### Statistical Analysis

The data were analyzed using R Studio (version 1.0.153, RStudio, Inc). For each diagnosis (eg, depression, anxiety, and substance use) we conducted an ANOVA test to determine whether the disorder-specific severity questionnaire scores were different among the 4 diagnostic groups (ie, “Yes”; “No, but I should be”; “I don’t know”; and “No”). We used Tukey post hoc tests to determine whether the differences in means between the groups were statistically significant.

## Results

### Descriptive Statistics

Table 2 presents the number of participants from each study who selected (1) “Yes”; (2) “No, but I should be”; (3) “I don’t know”; and (4) “No” to the question, “Have you ever been clinically diagnosed with [blank] disorder,” along with the percentage of the sample they represented. As shown in Table 2, study 1 had higher prevalence rates of each disorder assessed, indicating more clinical diagnoses in the national sample than in the college student sample. Missing data are not included in Table 2, but approximately 1% to 2% (from 11 to 19 participants in study 1 and 30 to 31 participants in study 2) did not answer the questions regarding diagnoses.

**Table 2:** Total number and percentage of participants who reported being diagnosed with mental disorders in a web-based panel (study 1; n=1123) and among university students (study 2; n=2337) who responded to a web-based survey on mental health and social media use (Survey Online Cohorts for Internalizing Symptoms and Language study).

Study and diagnosis			Yes, n (%)		No, but I should be, n (%)			I don't know, n (%)		No, n (%)
**Study 1 (n=1123)**										
	Insomnia	265 (23.64)		69 (6.16)			20 (1.78)		767 (68.42)	
	Somatic symptom disorder	58 (5.17)		5 (0.45)			31 (2.77)		1027 (91.61)	
	Depression	472 (42.11)		66 (5.89)			29 (2.59)		554 (49.42)	
	Social anxiety	292 (26.05)		64 (5.71)			39 (3.48)		726 (64.76)	
	Panic	207 (18.47)		43 (3.84)			26 (2.32)		845 (75.38)	
	Generalized anxiety	333 (29.71)		55 (4.91)			40 (3.57)		693 61.82)	
	Agoraphobia	65 (5.8)		20 (1.78)			37 (3.3)		999 (89.12)	
	Alcohol use disorder	114 (10.17)		16 (1.43)			26 (2.32)		965 (86.08)	
	Substance use disorder	117 (10.44)		12 (1.07)			26 (2.32)		966 (86.17)	
	Bipolar disorder	121 (10.79)		20 (1.78)			24 (2.14)		956 (85.28)	
**Study 2 (n=2337)**										
	Insomnia	138 (5.9)		208 (8.89)		46 (1.97)			1874 (80.09)	
	Somatic symptom disorder	23 (0.98)		14 (0.6)		45 (1.92)			2183 (93.29)	
	Depression	589 (25.17)		248 (10.6)		90 (3.85)			1340 (57.26)	
	Social anxiety	302 (12.91)		240 (10.26)		74 (3.16)			1649 (70.47)	
	Panic	140 (5.98)		65 (2.78)		56 (2.39)			2004 (85.64)	
	Generalized anxiety	663 (28.33)		324 (13.85)		75 (3.21)			1204 (51.45)	
	Agoraphobia	10 (0.43)		18 (0.77)		31 (1.32)			2206 (94.27)	
	Alcohol use disorder	12 (0.51)		16 (0.68)		11 (0.47)			2226 (95.12)	
	Substance use disorder	35 (1.5)		23 (0.98)		15 (0.64)			2192 (93.68)	
	Bipolar disorder	41 (1.75)		49 (2.09)		40 (1.71)			2135 (91.24)	

### Self-diagnosis Accuracy

#### Overview

In Table 3, we have presented the responses from the participants in studies 1 and 2 regarding the diagnoses of each disorder we assessed and whether they have been diagnosed: (1) “Yes”; (2) “No, but I should be”; (3) “I don’t know”; and (4) “No.” These have been presented along with the mean and SD of the disorder-specific severity questionnaire scores associated with each disorder. Table 3 also presents the results of a series of ANOVAs, followed by post hoc Tukey tests to examine potential differences in scores between the individuals who have been diagnosed (“Yes”) and those who selected “No, but I should be” compared with those who selected “No” or “I don’t know.”

If the disorder-specific severity questionnaire scores of individuals were “accurate” or consistent with their reported diagnostic status (“Yes”; “No, but I should be”; “I don’t know”; or “No”), we expected that the individuals who had not received a diagnosis would have lower mean scores than (1) those who had a diagnosis (“Yes”) and (2) those who said that they had no diagnosis but that they should be diagnosed (“No, but I should be”) and expected 3) that the “Yes” group and the “No, but I should be” group would be roughly comparable.

**Table 3:** Descriptive statistics and ANOVA results comparing reports of diagnosis across disorders.

Diagnosis (questionnaire: score range)		Yes, mean (SD)	NB^a^, mean (SD)	IDK^b^, mean (SD)	No, mean (SD)	Model, *F* test *(df)*	*P* value	Yes versus No, Tukey difference (range)	*P* value	NB versus No, Tukey difference (range)	*P* value	Yes versus NB, Tukey difference (range)	*P* value	
**Study 1 (n=1123)**														
	Insomnia (ISI^c^: 0 to 28)	16.39 (5.22)	15.61 (5.34)	13.25 (6.48)	9.56 (5.84)	107.2 (3,1105)	<.001	−6.84 (−7.89 to −5.73)	<.001	6.05 (4.21 to 7.89)	<.001	−0.80 (−2.77 to 1.19)	.73	
	Somatic symptom (SOM^d^: 0 to 30)	15.92 (7.64)	7.77 (9.29)	14.00 (5.66)	8.64 (6.39)	26.75 (3,1060)	<.001	−7.28 (−9.64 to −4.92)	<.001	−0.98 (−10.56 to 8.61);.99	.99	−8.26 (−18.09 to 1.58);.14	.14	
	Depression (PHQ^e^: 0 to 27)	12.13 (6.73)	11.98 (6.21)	10.38 (5.44)	5.85 (5.69)	92.23 (3,1098)	<.001	−6.27 (−7.27 to −5.27)	<.001	6.13 (4.06 to 8.20)	<.001	−0.14 (−2.22 to 1.95);.99	.99	
	Social anxiety (SAD^f^: 0 to 40)	18.38 (10.91)	13.58 (9.34)	14.72 (8.78)	7.81 (8.65)	90.39 (3,1092)	<.001	−10.57 (−12.26 to −8.88)	<.001	5.77 (2.66 to 8.88)	<.001	−4.80 (−8.10 to −1.50)	.001	
	Panic (PAN^g^: 0 to 40)	17.74 (10.56)	11.07 (8.17)	13.62 (10.71)	6.42 (8.83)	85.07 (3,1093)	<.001	−11.33 (−13.19 to −9.47)	<.001	4.65 (0.95 to 8.35)	.006	−6.68 (−10.65 to −2.70)	<.001	
	Generalized anxiety (GAD^h^: 0 to 40)	16.67 (10.46)	13.93 (7.62)	16.65 (10.36)	8.52 (9.41)	57.48 (3,1099)	<.001	−8.15 (−9.82 to −6.47)	<.001	5.41 (1.94 to 8.87)	<.001	−2.74 (−6.35 to 0.86)	.20	
	Alcohol use (AUDIT^i^: 0 to 40)	15.97 (10.07)	8.44 (5.60)	14.15 (11.41)	4.96 (7.29)	77.73 (3,1097)	<.001	−11.33 (−13.00 to −9.03)	<.001	3.48 (−1.52 to 8.8)	.28	−7.54 (−12.84 to −2.23)	.002	
	Drug use (SUD^j^: 0 to 40)	11.05 (9.80)	7.08 (6.79)	14.46 (11.52)	2.65 (5.59)	84.36 (3,1096)	<.001	−8.40 (−10.00 to −6.79)	<.001	4.43 (−.14 to 8.98)	.06	−3.97 (−8.75 to 0.80)	.14	
	Bipolar (PHQ: 0 to 27)	15.06 (7.03)	12.95 (5.22)	11.92 (6.49)	8.04 (6.48)	44.88 (3,1092)	<.001	−7.02 (−8.66 to −5.38)	<.001	4.91 (1.12 to 8.70)	.004	−2.11 (−6.16 to 1.94)	.54	
	Bipolar (ASRM^k^: 0 to 20)	11.79 (5.54)	9.95 (4.39)	13.43 (4.88)	11.06 (5.54)	2.75 (3,1097)	.04	−0.72 (−1.97 to 0.52)	.43	−1.11 (−3.95 to 1.73)	.75	−1.84 (−4.89 to 1.20)	.40	
**Study 2 (n=2337)**														
	Insomnia (ISI: 0 to 28)	14.20 (5.61)	14.56 (4.61)	12.96 (5.99)	8.07 (4.89)	170.4 (3,2261)	<.001	−6.13 (−7.25 to −501)	<.001	6.49 (5.56 to 7.42)	<.001	0.36 (−1.03 to 1.75)	.91	
	Somatic symptom (SOM: 0 to 30)	12.95 (6.56)	11.59 (6.08)	11.45 (5.42)	6.92 (4.68)	31.00 (3,2164)	<.001	−6.04 (−8.60 to −3.48)	<.001	5.51 (2.24 to 8.78)	<.001	−0.52 (−4.66 to 3.60)	.98	
	Depression (PHQ: 0 to 27)	10.58 (6.08)	11.59 (6.08)	7.46 (4.94)	5.00 (4.34)	232.40 (3,2259)	<.001	−5.58 (−6.22 to −4.93)	<.001	6.59 (5.9 to 7.49)	<.001	1.01 (0.03 to 2.00)	.04	
	Social anxiety (SAD: 0 to 40)	15.74 (9.68)	15.15 (8.96)	10.29 (6.08)	5.63 (6.18)	265.30 (3,2255)	<.001	−10.11 (−11.24 to −8.97)	<.001	9.51 (8.25 to 10.77)	<.001	−0.60 (−2.17 to 0.98)	.76	
	Panic (PAN: 0 to 40)	11.89 (8.84)	10.98 (8.38)	9.22 (8.68)	2.74 (4.68)	194.60 (3,2253)	<.001	−9.15 (−10.34 to −7.96)	<.001	8.24 (6.53 to 9.96)	<.001	−0.91 (−2.95 to 1.13)	.66	
	Generalized anxiety (GAD: 0 to 40)	13.21 (8.57)	11.57 (7.25)	11.03 (7.28)	5.53 (5.26)	208.30 (3,2048)	<.001	−7.67 (−8.51 to −6.83)	<.001	6.03 (4.94 to 7.12)	<.001	−1.64 (−2.82 to −0.46)	.002	
	Alcohol use (AUDIT: 0 to 40)	11.50 (8.54)	16.88 (9.81)	11.03 (7.28)	5.53 (5.26)	54.51 (3,2132)	<.001	−6.61 (−10.02 to −2.99)	<.001	11.98 (8.85 to 15.12)	<.001	5.38 (0.61 to 10.14)	.02	
	Drug use (SUD: 0 to 40)	3.14 (2.05)	3.43 (1.83)	2.40 (1.76)	0.70 (1.45)	63.89 (3,2260)	<.001	−2.44 (−3.08 to −1.80)	<.001	2.74 (1.95 to 3.52)	<.001	0.29 (−0.72 to 1.30)	.88	
	Bipolar (PHQ: 0 to 27)	11.80 (5.78)	12.98 (6.24)	11.69 (5.57)	6.97 (5.65)	35.33 (3,2257)	<.001	−4.84 (−7.13 to −2.54)	<.001	6.01 (3.91 to 8.12)	<.001	1.17 (−1.91 to 4.26)	.76	
	Bipolar (ASRM: 0 to 20)	4.86 (2.54)	6.83 (2.59)	4.80 (3.29)	4.80 (3.40)	1.43 (3,659)	.23	−0.05 (−2.39 to 2.29)	.99	2.03 (−0.50 to 4.56)	.16	1.98 (−1.43 to 5.39)	.44	

^a^NB: “No, But I should be.”

^b^IDK: “I don’t know.”

^c^ISI: Insomnia Severity Index.

^d^SOM: severity measure for somatic symptoms—adult.

^e^PHQ: Patient Health Questionnaire (Severity Measure of Depression).

^f^SAD: Severity Measure for Social Anxiety Disorder.

^g^PAN: severity measure for panic disorder.

^h^GAD: severity measure for generalized anxiety disorder.

^i^AUDIT: Alcohol Use Disorders Identification Test.

^j^SUD: level 2—Substance Use—Adult.

^k^ASRM: Altman Self-rating Mania Scale.

#### Study 1

For most conditions assessed, the results were consistent with our expectations, indicating that the mean disorder-specific severity questionnaire scores for the four diagnostic conditions—(1) “Yes”; (2) “No, but I should be”; (3) “I don’t know”; and (4) “No”—were different between the groups. For self-reported mania, somatic symptom disorder, and alcohol use disorder, the pattern of mean differences did not suggest that the individuals were accurate in their assessment. However, as indicated by ANOVAs and post hoc Tukey tests, for social anxiety disorder, panic disorder, alcohol use disorder, substance use disorder, and bipolar disorder, there were significant differences (*P*<.05) in questionnaire scores between the individuals who had been previously diagnosed by a clinician and those who believed that they should be diagnosed. We were expecting the individuals who selected “Yes” and those who selected “No, but I should be” to present similar scores, but, instead, the individuals who reported they had a diagnosis had higher questionnaire scores, indicting greater disorder severity. The ASRM appears to be a poor measurement tool for the self-diagnosis of bipolar disorder: it did not differentiate the individuals who had been diagnosed from those who had not. However, when using the PHQ-9 to assess the depressive symptoms in bipolar disorder, the scores on the PHQ-9 were different in the expected directions between the groups.

#### Study 2

In all disorders but bipolar disorder, the ANOVA results were consistent with our expectations, indicating that the mean disorder-specific severity questionnaire scores between the 4 diagnosis conditions—(1) “Yes”; (2) “No, but I should be”; (3) “I don’t know”; and (4) “No”—were different between the groups. Upon further investigation with post hoc Tukey tests, there were no differences in disorder-specific severity questionnaire scores between the individuals who had been previously diagnosed by a clinician and those who believed that they should be diagnosed with insomnia, somatic symptom disorder, social anxiety disorder, panic disorder, or GAD. There were significant differences between previously diagnosed individuals and those who believed that they should be diagnosed with depression, GAD, or alcohol use disorder. Here, the depression and alcohol use scores were actually higher in the group of people who believed that they should be diagnosed, potentially indicating stigma around reporting to a professional or difficulty accessing care. Of note, a smaller portion of our sample in study 2 completed the ASRM, as it was removed partway through the study owing to its poor ability to capture self-reported mania. The ASRM appeared to poorly capture the differences in mania symptoms among the individuals who have been diagnosed, those who believed that they should be diagnosed, and those who were not been diagnosed, suggesting that a self-report assessment of mania may not be a good barometer of bipolar-spectrum psychopathology in college students. However, the PHQ-9 was able to detect differences among the groups in the expected directions for bipolar disorder.

## Discussion

### Principal Findings

This study investigated the scores on self-reported disorder-specific severity questionnaires of individuals who indicated that they had been previously diagnosed (“Yes”), believed that they should be diagnosed (“No, but I should be”), were unsure (“I don’t know”), and had never been diagnosed (“No”) across a variety of internalizing and externalizing disorders. Given the misdiagnosis, underdetection, and stigma associated with mental disorders, this study was a novel way to explore the “accuracy” of self-diagnosis in a national survey panel and a sample of college students. Overall, the results indicate that people who say they have been diagnosed with a disorder have the highest severity scores in general, but this is closely followed by individuals who have not been diagnosed but believe that they should be. This is especially true for clinical conditions that are relatively nonspecific and whose names are relatively descriptive such as depression, generalized anxiety, and insomnia. The results suggest that self-reported mania questionnaires do not align with reported diagnoses, which is consistent with prior studies [33-35], although there is some evidence that self-reported measures can correlate with a lifetime diagnosis of bipolar disorder [36]. Additional differences in patterns observed between our samples contribute to our interpretation of these findings; with some exceptions, college students tended to be more accurate than the national sample in self-diagnosis.

Although study 2 consisted of college students in the Midwestern United States, which was a relatively homogeneous population in terms of age and ethnicity, our national sample (study 1) was more variable in age, ethnicity, and other demographic characteristics, including age and gender. Our national sample from study 1 also reported more severe psychopathology. The differences between our samples, with college students being slightly more accurate in self-diagnosis, yield additional support for the validity of university samples, which are often criticized for lacking generalizability [37]. Initially, we had expected that college students may not know as much about their diagnoses, but it seems that college students are reasonably accurate in self-diagnosis, reflecting trends of reduced stigma and increased awareness of the mental health concerns that are present in early adulthood [38,39]. In addition, it is possible that the college students were more accurate in self-diagnosis because they were psychology students and, therefore, were aware of various mental health complaints. A secondary interpretation is that younger adults are generally better at web-based help-seeking behaviors and thus more attuned to their personal mental states [40]. Indeed, younger adults may be more prone to and more savvy with engaging in web-based information seeking, including the use of social media support networks to understand their mental health status [41]. This makes social media a useful tool for studying mental health as well as disseminating support resources, treatment options, and research.

### Comparison With Prior Work

Overall, our hypotheses were supported. Our findings showed that self-diagnosis is accurate for internalizing disorders, meaning that most individuals with disorders such as major depression and GAD can accurately detect that their symptoms are significant, even if they have not received a formal diagnosis. Our findings also reveal that self-reported diagnoses are more accurate for more commonly occurring conditions; for example, depression and GAD showed high accuracy in self-diagnosis, but they are also among the most common mental disorders [42,43], unlike other anxiety disorders like panic that occur at lower frequencies [43,44]. It is possible that the accuracy of self-diagnosis corresponds best to disorders that occur at high base rates, which could partially explain our poor self-diagnosis accuracy for mania, panic, and somatic symptom disorders. Indeed, prior research has demonstrated that self-reporting is more accurate for people who are more familiar with a disorder and its symptoms [45]. Social media may play an important role in disseminating information about common mental health disorders to communities of interest such as adolescents and young adults.

Interestingly, consistent with prior work, the participants in both samples showed poor abilities in detecting mania symptoms via self-report compared with diagnoses from professionals. We acknowledge this is not the first study to find that mania is more difficult to detect than depressive symptoms in a self-report form [33]. It is likely that bipolar disorder, specifically manic symptoms, cannot be screened via self-report, and a multistage screener with clinician report may be necessary [33-35]. It is also possible that the time frame of the ASRM assessment, that is, the past week, does not align well with the diagnosis of bipolar disorder, for which a manic episode could have occurred years ago [46]. Indeed, for most patients with a bipolar-spectrum illness, mania symptoms are rarely present at the time of the assessment.

Alcohol and substance use disorders followed bipolar disorder in terms of lowest self-diagnosis accuracy. We offer several explanations for this finding. First, in college students, alcohol consumption, particularly binge drinking, is much more prevalent than in the general public [47,48], and the behavior is normalized and encouraged in many social groups [49,50]. This may lead to a low likelihood of reporting interference from drinking, despite a high level of alcohol use. However, this is likely not the case in our studies, as in our college student sample, the participants who believed that they should be diagnosed actually had higher AUDIT scores than those who had already been diagnosed, indicating good insight. However, given that the rates were higher in those who believed that they should be diagnosed than in those who had received diagnoses, it is possible that college students who are struggling with alcohol and drug problems receive diagnoses for other comorbid conditions (depression) and do not receive substance use disorder diagnoses until they are out of college when heavy drinking behaviors are not normalized. Indeed, in our college student sample, <1% had been diagnosed with an alcohol use disorder, whereas in the national sample, approximately 10% had previously received a diagnosis, which is in line with epidemiological data [51]. In our national sample, which had lower socioeconomic status and may have had lower educational attainment overall compared with our university sample, there was a lower correspondence between self-diagnosis and self-report disorder-specific severity questionnaire scores, indicating a need for further research into drinking and substance use behaviors, perhaps via more daily monitoring of use.

### Limitations

Despite the strengths of our study, including its novelty and the use of 2 large samples, it is not without its limitations. First, we did not have clinicians interview the participants to assess their disorders at the time of the study. The reported disorders were based on prior clinical diagnoses and relied on participants’ honesty and awareness of their mental health conditions. Moreover, we did not include an assessment of all disorders, including those that may resemble mood disorders early in their course, including psychotic disorders, personality disorders, and medical or substance-induced disorders, as we focused most thoroughly on the internalizing spectrum. Future research should consider assessing the full spectrum of psychopathology. In addition, we note that the timeline of disorder onset is not clear from our questionnaires. Some participants may have received their diagnoses years before the current assessment period, and our questionnaires asked about their symptoms within the last week or 2 weeks. Second, differential diagnosis was nearly impossible in our sample, given the wide variety of disorders and lack of clinical reports. This means that the participants may have appeared to have a higher degree of psychopathology than was truly representative, given their diagnostic comorbidities, *DSM* hierarchy rules, and rule-outs that clinicians would be aware of but patients may not be aware of. Third, it is possible that in our study, the participants appeared to be good at yielding accuracy in the self-diagnoses of some disorders such as social anxiety disorder, but this is a reflection of the measure being highly sensitive but not specific. Further testing of the *DSM* severity measures in a wide variety of samples is warranted before their extended use.

### Conclusions

Our results support ongoing and future research that uses patient self-report of symptoms, diagnoses, and disorders on a more solid footing. In particular, for some conditions of common mental disorders such as depression, anxiety, and insomnia, relying on textual reports may not bias samples, at least in terms of symptom severity: when individuals say that they struggle with a problem, they in fact seem to do so. The next steps of this research involve examining these results in conjunction with relevant features of individuals’ social media accounts, including sentiment analyses, text analysis of themes associated with internalizing disorders, and other computational informatics analyses of social media data. Indeed, we have previously shown that individuals who self-disclose depression diagnoses use more cognitive distortions [16], have different circadian rhythms [15], and show more negative affect based on sentiment analysis according to unpublished data from Rutter et al (Rutter, LA, unpublished data, August 2022). However, the validity of results from Twitter have been called into question because self-disclosure is not well understood and is often stigmatized as a nonefficacious source of data [52]. It is also possible that individuals who self-disclose their diagnoses on the web are different from those who report their diagnoses and symptoms via survey. On the basis of the results of this study, we can now more definitively say that self-reported survey diagnoses correspond well with symptom severity on a continuum and can, on balance, be trusted as clinical indicators, especially in internalizing disorders such as depression and GAD. However, more research on the validity and modalities of web-based self-reports is needed, and research should be expanded to capture other related mental disorders. Gaps in research notwithstanding, if more studies ask about self-reported diagnoses and compare self-reported symptoms, the field could benefit by receiving more accurate detection rates. In general, if a person believes that they should be diagnosed with an internalizing disorder, they may be experiencing a degree of psychopathology that is similar to that experienced by someone who has been diagnosed, warranting clinical attention. Psychologists and providers can put more confidence into the patient’s perspective of their disorders and when time is limited, reduce questionnaire questions and query more specifically if the person believes that they have a disorder. In addition, researchers may be able to place more trust in social media data when individuals report diagnoses of anxiety and depression; however, replication and further study are needed. This perspective may yield additional insight into the burden of mental illness and the associated distress and impairment.

## Abbreviations

APA: American Psychiatric Association
ASRM: Altman Self-rating Mania Scale
AUDIT: Alcohol Use Disorders Identification Test
DSM: Diagnostic and Statistical Manual for Mental Disorders
GAD: generalized anxiety disorder
PHQ-9: Patient Health Questionnaire-9
